# Developmental Validation of a Rapidly Mutating Y-STR Panel Labeled by Six Fluoresceins for Forensic Research

**DOI:** 10.3389/fgene.2022.777440

**Published:** 2022-03-03

**Authors:** Xiaoye Jin, Hongling Zhang, Zheng Ren, Qiyan Wang, Yubo Liu, Jingyan Ji, Han Zhang, Meiqing Yang, Yongsong Zhou, Jiang Huang

**Affiliations:** ^1^ Department of Forensic Medicine, Guizhou Medical University, Guiyang, China; ^2^ Guangzhou Key Laboratory of Forensic Multi-Omics for Precision Identification, School of Forensic Medicine, Southern Medical University, Guangzhou, China

**Keywords:** Y-STR, male differentiation, developmental validation, forensic research, rapidly mutating

## Abstract

The male-specific region of the human Y chromosome is a useful genetic marker for genealogical searching, male inheritance testing, and male DNA mixture deconvolution in forensic studies. However, the Y chromosomal short tandem repeats (Y-STRs) are difficult to distinguish among related males due to their low/medium mutation rate. In contrast, rapidly mutating (RM) Y-STRs exhibit unusually high mutation rates and possess great potential for differentiating male lineages. In this study, we developed a novel Y-STRs multiplex amplification assay of 32 RM Y-STRs by fragment analysis using six dye-labeled technologies (FAM, HEX, TAMRA, ROX, VIG, and SIZ). The development and the validation of the kit were carried out in accordance with the Scientific Working Group guidelines on DNA Analysis Methods. Identical allelic profiles of the 32 RM Y-STRs using a DNA 9948 sample as the positive control could be observed at different concentrations of PCR reagents. Further, the RM Y-STRs did not show cross-reactions with other common animal species, and the developed assay could tolerate interferences from common PCR inhibitors and mixed DNA samples. More importantly, the kit showed relatively high sensitivity and could detect trace DNA samples. Genetic distributions of 32 RM Y-STRs in the Guizhou Han population revealed that these RM Y-STRs showed relatively high genetic diversities. In conclusion, the RM Y-STR assay developed here showed good species specificity, high sensitivity, tolerance to inhibitors, and sample compatibility, which can be viewed as a highly efficient tool with high discrimination capacity for forensic male differentiation.

## Introduction

The majority of the human Y chromosome regions does not recombine and thus exhibit a strict male inheritance pattern from father to son (Kayser, 2017). Genetic markers on the Y chromosome serve as powerful tools that are widely employed in forensics to determine the lineage of the male pedigree and perform DNA mixture deconvolution of forensic samples related to sexual assaults (Ruitberg et al., 2001). Short tandem repeat (STR) loci exhibit alleles defined by variable numbers of short sequence motifs and can possess many allele variations in populations that lead to extremely high genetic diversities. In combination, STRs found on the Y chromosome can provide male identification, pedigree searching, and paternity analysis, and thus, commercial Y-STR kits have been developed. For example, the Yfiler plus kit (Thermo Fisher Scientific, Waltham, MA, United States) can simultaneously amplify 27 Y-STRs, which together provide relatively high discrimination capacity (DC) (Fan et al., 2021a); the Microreader Y Prime Plus ID panel (Microread Genetics, Suzhou, Jiangsu, China) comprising a Y chromosome insertion/deletion variant and 37 Y-STRs was developed for the construction of the Chinese DNA database (Zhao et al., 2021). In some populations, existing Y-STRs are not variable enough to differentiate closely related males, or even distantly related males, because most Y-STR loci in the available kits have relatively low mutation rates and therefore exhibit relatively few alleles.

Y-STR loci exhibiting higher mutation rates, the so-called rapidly mutating (RM) Y-STR loci, have been identified that have the potential to provide more discriminative power to distinguish related males. Ballantyne et al. (2010) investigated the mutation rates of 186 Y-STRs in a large sample of father–son pairs, identifying 13 RM Y-STRs with mutation rates on the order of 1 in 100 gametes. In follow-up studies, these 13 RM Y-STRs exhibited higher male differentiation efficiency for closely related men than the Y-STR loci found in commercial kits (Ballantyne et al., 2012; Ballantyne et al., 2014; Alghafri et al., 2015; Adnan et al., 2016). Ralf et al. (2020) screened 27 candidate RM Y-STRs *via* an *in silico* approach and pinpointed 12 novel RM Y-STRs in a large number of male pedigrees. Zhang et al. (2017) designed a multiplex panel of 13 RM Y-STRs and evaluated their mutation rates in Chinese Han populations, showing that nearly 20% of father–son pairs could be differentiated by these loci. Zhou et al. (2021) reported a validation study of a multiplex amplification system including 17 RM Y-STRs with high DC in Chinese male individuals. These RM Y-STR loci could expand the power of male identification if commercially available as a kit.

In this study, we reported the development and validation of a novel Y-STR multiplex PCR amplification panel, HomyGene RM Y32 (HomyGene, Foshan, Guangdong, China), which co-amplifies 32 RM Y-STRs that can be genotyped by fragment analysis using six dye-labeled technology (FAM, HEX, TAMRA, ROX, VIG, and SIZ). Following the guidelines published by the Scientific Working Group on DNA Analysis Methods (Methods, 2016), we evaluated this novel RM Y-STR panel to assess its performance in genotyping accuracy and precision, sensitivity, and the power to discriminate males in DNA mixtures (deconvolution). We also evaluated species specificity, different sample compatibility, and PCR performance when samples contained a number of possible inhibitor compounds.

## Materials and Methods

### Sample Information

We used a 9948 DNA (1 ng/μl) sample to evaluate PCR amplification conditions, accuracy, DNA mixture deconvolution, and inhibitor tolerance. Further, a sample containing 9947A DNA was used to assess the male specificity of the HomyGene RM Y32 kit. DNA samples (1 ng/μl) from two unrelated male individuals were used to perform the mixture analysis. Common bacterial and animal species, including colibacillus, chicken, pig, dog, sheep, cat, and cow, were chosen to evaluate the species specificity of the kit. Mock forensic case-type samples including hair root, blood stain (wall), saliva stain (table top), semen stain (underpants), and cell phone swabs were obtained from seven anonymous males. For the hair root samples, we obtained three hair roots from each individual. In addition, we collected samples from 232 unrelated healthy Han individuals living in the Guizhou province, China. All individuals in this study provided their written informed consent for participation. This research conformed to the guidelines and obtained the approval of the ethics committee of Guizhou Medical University.

### Loci Selection and Primer Design

RM Y-STRs that showed high mutation rates or genetic diversities were chose from previous studies (Huang et al., 2005; Lin et al., 2006; Ballantyne et al., 2010, 2012; Zhang et al., 2014; Meng et al., 2019; Liu et al., 2020a). Next, the primer pair for each RM Y-STR locus was designed using the Primer 3 online tool (Untergasser et al., 2012) and was synthesized by the Sangon Biotech (Shanghai, China).

### PCR Amplification, Capillary Electrophoresis, and Data Analysis

All experiments were conducted according to the following parameters unless stated otherwise. The PCR cocktail consisted of 10 μl of reaction mix (HomyGene), 5 μl of RM Y32 primer mix (HomyGene), 1 μl of C-Taq polymerase (HomyGene), 1 μl of DNA sample (1 ng/μl), and 8 μl of sdH_2_O. Multiplex PCR of each sample was performed on the ProFlex PCR system (Thermo Fisher Scientific). The detailed reaction conditions were as follows: 95°C for 2 min, 10 cycles of 94°C for 30 s, 58.5°C for 60 s, and 72°C for 60 s; 20 cycles of 90°C for 30 s, 57°C for 60 s, and 72°C for 60 s; and 72°C for 10 min.

PCR product (1 μl volume) was added to the mixture containing 10 μl of deionized HiDi Formamide (Thermo Fisher Scientific) and 0.3 μl of AGCU Marker SIZ-500 (AGCU ScienTech Incorporation, Wuxi, Jiangsu, China). We performed capillary electrophoresis using the 3500xL Genetic Analyzer (Thermo Fisher Scientific) equipped with 36-cm capillary arrays with POP-4^®^ Polymer (Thermo Fisher Scientific). First, spectral calibration was conducted on the 3500xL Genetic Analyzer using the HomyGene Six Dye Matrix Standard kit (HomyGene). Next, we separated and detected amplified products of 32 RM Y-STRs on the 3500xL Genetic Analyzer. GeneMapper^®^ ID-X Software v1.5 (Thermo Fisher Scientific) was used to assess allele typing of each locus by comparison with the allelic ladder. The 150 relative fluorescence unit (RFU) was used as the analytical threshold to detect allele peak, unless stated otherwise.

The allelic ladder for this assay was constructed according to the previous study (Jia et al., 2021). In summary, a series of DNA samples, which showed different allelic variations of 32 RM Y-STRs in population, were sequentially amplified using the designed primer pairs. For each RM Y-STR locus, the amplified product of each allele was collected, purified, and mixed to obtain an allelic ladder of the locus. Then, the allelic ladder of each RM Y-STR locus was mixed in reasonable proportions to produce the allelic ladder for the kit.

### PCR-Based Study

We used a 1 ng 9948 DNA sample to perform PCR in the presence of different concentrations of amplification reagents or various reaction conditions to determine the optimal reaction parameters of the kit. Only the index to be tested was changed, and other parameters remained fixed. Each test was conducted in triplicate. The testing conditions are listed as below (the parameter in bold was the recommended condition):

Annealing temperatures: 57.5°C and 56°C, 58°C and 56.5°C, **58.5**°C and **57**°C, 59°C and 57.5°C, and 59.5°C and 58°C.

PCR Cycles: 28, **30**, and 32 cycles
Primer mix: 3.75, **5**, and 6.25 μl
Reaction mix: 7.5, **10**, and 12.5 μl
C-Taq polymerase: 0.75, **1**, and 1.25 μl

### Accuracy, Size Precision, and Sample Adaptability Testing

The 1 ng 9948 DNA sample was amplified and detected by three different operators to validate the genotyping accuracy of the kit. In addition, 10 bloodstain samples were randomly selected and subjected to multiplex PCR amplification and allelic typing using the AGCU Y37 kit (AGCU ScienTech Incorporation) to assess the genotype concordances across different kits. The allelic ladder was injected and tested on the 3500xL Genetic Analyzer 24 times to analyze the precision of the allele size.

Different bloodstain cards including FTA, Haoyuan, Kecaifeng reinforce, Kecaifeng mini, Dabo, Xinhai, Xinhai 503, filter paper, Bokun classic, Bokun reinforce, and Bokun mini cards were used to store blood samples from the same individual. The storage time of these bloodstain cards ranged from 3 to 5 years. These cards were assessed by the developed system to evaluate sample compatibility.

The DNA samples of mock case-type samples were extracted by the EZ1 DNA Investigator kit (Qiagen, Hilden, Germany). Next, these DNA samples were amplified by the developed system. Finally, amplified products were separated and typed as indicated above, respectively.

### Mixture and Sensitivity Studies

The 9948 and 9947A DNA samples were mixed in different ratios (19:1, 9:1, 3:1, 1:1, 1:3, 1:9, and 1:19) to evaluate the male specificity of the kit. Next, DNA samples from two unrelated males were mixed in different ratios (19:1, 9:1, 3:1, 1:1, 1:3, 1:9, and 1:19) to evaluate the power of the kit to discriminate the mixture. The mixtures were amplified in triplicate using the developed kit. For these mixtures, the 50 RFU was viewed as the analytical threshold to detect allele peak.

The control sample 9948 (1 ng/μl) was serially diluted into 1, 0.5, 0.25, 0.125, and 0.0625 ng. Next, these samples, in triplicate, were used to evaluate the detection limit of the kit.

### Species Specificity and Stability Study

A 1 ng DNA sample from different species was used to assess the cross-reaction of the kit. Each test was conducted three times.

Frequently encountered PCR inhibitors were selected to evaluate the stability of the kit. These inhibitors were heme (300, 400, 500, and 600 μM), hemoglobin (50, 100, 200, and 260 μM), humic acid (50, 150, 200, and 260 ng/μl), indigo (12, 14, 17, and 20 mM), Ca^2+^ (1.0, 1.5, 2.0, and 2.6 mM), and EDTA (1.0, 1.5, 2.0, and 2.5 mM). Each inhibitor was tested in triplicate.

### Statistical Analysis

Allelic frequencies and gene diversities (GDs) of 32 RM Y-STRs in Guizhou Han population were estimated using the STRAF online tool (Gouy and Zieger, 2017). Haplotype match probability (HMP), DC, and haplotype diversity (HD) of 32 RM Y-STRs in the Guizhou Han population were calculated on the basis of a previous study (Liu et al., 2020b).

## Results and Discussion

### Loci Information

**FIGURE 1:**
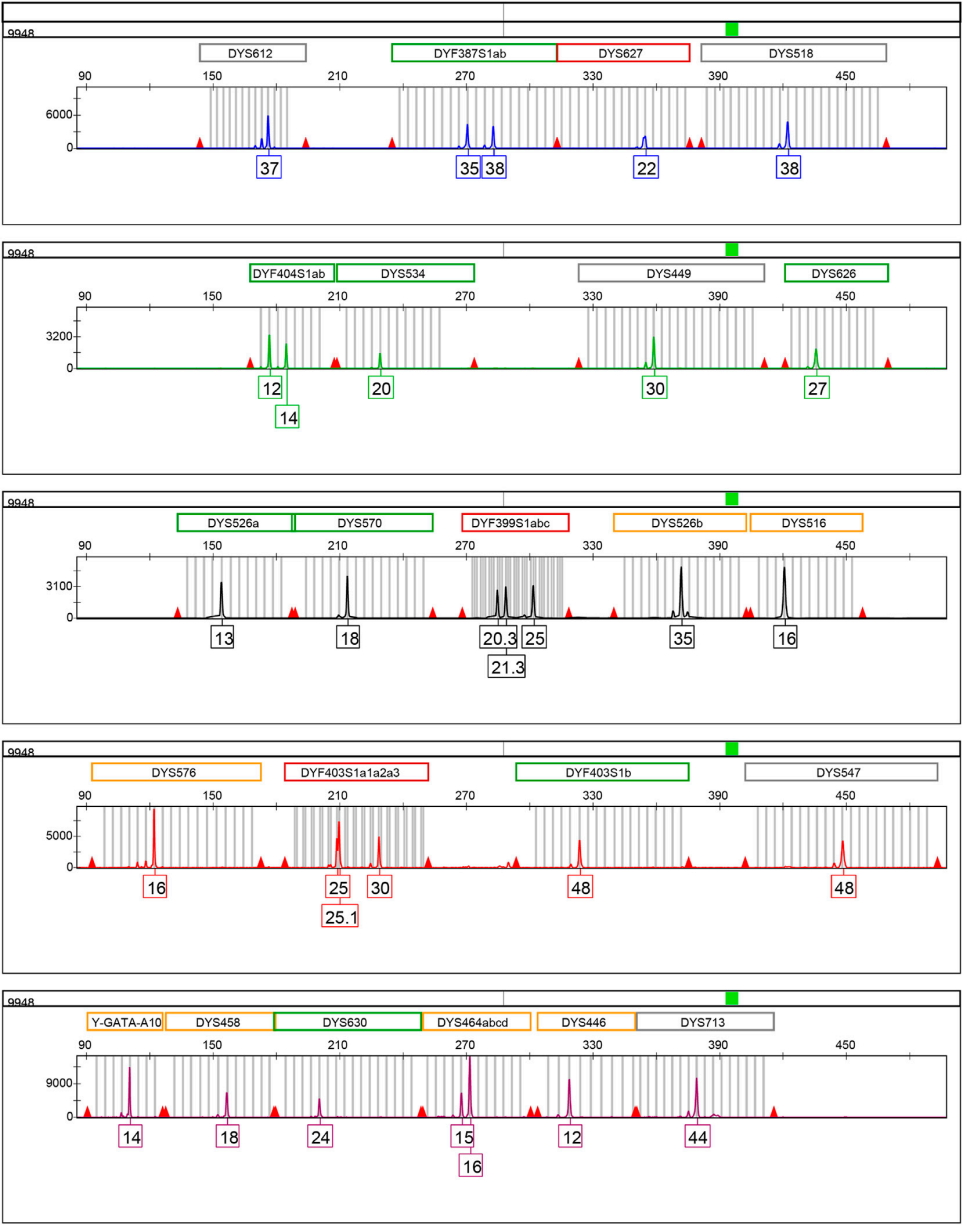
Allelic profile of 32 RM Y-STRs of the positive control DNA sample 9948 (1 ng/μl) amplified by the HomyGene RM Y32 panel. Different colors represent different dyes: blue for FAM, green for HEX, black for TAMRA, red for ROX, and purple for VIG.

The information relative to the 32 RM Y-STR loci is provided in Table 1. These 32 loci were classified into five sub-groups labeled by different dyes: DYS612, DYF387S1a/b, DYS627, and DYS518 (FAM); DYF404S1a/b, DYS534, DYS449, and DYS626 (HEX); DYS526a/b, DYS570, DYF399S1a/b/c, and DYS516 (TAMRA); DYS576, DYF403S1a1/a2/a3, DYF403S1b, and DYS547 (ROX); Y-GATA-A10, DYS458, DYS630, DYS464a/b/c/d, DYS446, and DYS713 (VIG). The amplicon ranges of these 32 RM Y-STRs are illustrated in Supplementary Figure S1. The amplicon lengths of these RM Y-STRs in the developed kit ranged from 90 to 500 base pairs (bp). The allelic profile of the 9948 DNA sample for these 32 RM Y-STRs is shown in Figure 1.

**TABLE 1:** Loci information of 32 RM Y-STRs.

Loci	Repeat motif	Chromosomal location	Allele range	Amplicon length (bp)	Dye	Mutation rate	References
DYS612	CCT, CTT, TCT	Yq11.221	28–40	145–195	FAM	1.45 × 10^–2^	Ballantyne et al. (2012)
DYF387S1a/b	AAAG, GTAG, GAAG	Yq11.223	28–45	236.5–313.5	FAM	1.59 × 10^–2^	Ballantyne et al. (2012)
DYS627	AGAG, AAAG	Yp11.2	10–28	314–378	FAM	1.23 × 10^–2^	Ballantyne et al. (2012)
DYS518	AAAG, GAAG, GGAG	Yq11.221	30–49.2	379.5–470	FAM	1.84 × 10^–2^	Ballantyne et al. (2012)
DYF404S1a/b	TTTC	Yq11.23	8–19	169–209	HEX	1.25 × 10^–2^	Ballantyne et al. (2012)
DYS534	CTTT	Yq11.221	16–27	210–275	HEX	1.07 × 10^–2^	Fan et al. (2021b)
DYS449	TTTC	Yp11.2	21–42	324–414	HEX	1.22 × 10^–2^	Ballantyne et al. (2012)
DYS626	GAAA, GAAG	Yq11.223	23–35	420–470	HEX	1.22 × 10^–2^	Ballantyne et al. (2012)
DYS526a	CCTT, CTTT, CCTT	Yp11.2	9–20	130–185	TAMRA	1.25 × 10^–2^	Ballantyne et al. (2012)
DYS526b	CCTT, CTTT, CCTT	Yp11.2	28–42	333–397.5	TAMRA	1.25 × 10^–2^	Ballantyne et al. (2012)
DYS570	TTTC	Yp11.2	10–28	188–255	TAMRA	1.24 × 10^–2^	Ballantyne et al. (2012)
DYF399S1a/b/c	GAAA	Yq11.223	17.3–28.1	268–318	TAMRA	7.73 × 10^–2^	Ballantyne et al. (2012)
DYS516	TTCT	Yq11.221	13–24	407–460	TAMRA	0.39 × 10^–2^	Fan et al. (2021b)
DYS576	AAAG	Yp11.2	10–27	94–172	ROX	1.43 × 10^–2^	Ballantyne et al. (2012)
DYF403S1a1/a2/a3	TTCT	Yp11.2	20–35.1	186–254	ROX	3.10 × 10^–2^	Ballantyne et al. (2012)
DYF403S1b	TTCT, TTCC	Yp11.2	43–60	296–377	ROX	1.19 × 10^–2^	Ballantyne et al. (2012)
DYS547	TTCC, TTTC	Yq11.221	38–58	405–496	ROX	2.36 × 10^–2^	Ballantyne et al. (2012)
Y-GATA-A10	TATC	Yq11.221	9–19	91–127	VIG	0.09 × 10^–2^	Fan et al. (2021b)
DYS458	GAAA	Yp11.2	10–24	127.5–178.5	VIG	0.49 × 10^–2^	Fan et al. (2021b)
DYS630	AAAG, GAGA, AAGA, AGAG	Yq11.222	23–35	180–250	VIG	1.26 × 10^–2^	Fan et al. (2021b)
DYS464a/b/c/d	CCTT	Yq11.223	-	251–302	VIG	0.22 × 10^–2^	Fan et al. (2021b)
DYS446	TCTCT	Yp11.2	10–20	305–351	VIG	0.09 × 10^–2^	Fan et al. (2021b)
DYS713	TCTT, CTTT, TTCT, CTTTT, TTAT	Yp11.2	36–52	351.5–418	VIG	1.17 × 10^–2^	Fan et al. (2021b)

For these 32 RM Y-STRs, more than half of Y-STRs were those RM Y-STRs (DYF387S1, DYF399S1, DYF403S1, DYF404S1, DYS449, DYS518, DYS526, DYS547, DYS570, DYS576, DYS612, DYS626, and DYS627) identified by Ballantyne et al. (2010). Subsequently, the same research group also evaluated differentiation efficiencies of these RM Y-STRs in different continental and endogamous populations, and they found that these loci showed a higher male DC than the 17 Y-STRs from the Y filer kit (Thermo Fisher Scientific) (Ballantyne et al., 2012; Adnan et al., 2016). Zhang et al. assessed the mutation rates of these loci in the Hubei Han population and stated that their mutation rate ranged from 2.00 × 10^–3^ to 4.59 × 10^–2^ (Zhang et al., 2017). Yuan et al. (2019) also investigated the genetic distributions of these RM Y-STRs in the Beijing Han population, and they discerned similar results. For the remaining loci (DYS446, DYS458, DYS464, DYS516, DYS534, DYS630, DYS713, and Y-GATA-A10), Feng et al. (2020) found that DYS446, DYS458, DYS630, and Y-GATA-A10 displayed relatively high GD values (>0.5) in four minority groups from China. Zhou et al. (2021) discovered that mutation rates of DYS464 and DYS713 loci in the Sichuan Han population were 4 × 10^–3^ and 2.4 × 10^–2^, respectively. Fan et al. (2021b) reported that mutation rates of DYS516 and DYS534 loci in the coastal southeastern Han populations were 3.89 × 10^–3^ and 1.07 × 10^–2^, respectively. In summary, these 32 RM Y-STRs loci showed relatively high mutation rates and GD values across Chinese populations and could be beneficial in differentiating male lineages and unrelated males. However, we did not investigate the mutation rates of these 32 RM Y-STRs in the current study.

### PCR-Based Study

In practice, the annealing temperature may be affected by variations of the thermal cycler. Accordingly, it is essential to assess the effect of temperature variation on the Y-STR profile. For the HomyGene RM Y32 panel, two thermal cycle reactions are needed that possess different reaction conditions. Therefore, we simultaneously assessed the effect of annealing temperature 1 (first thermal cycle reaction) and annealing temperature 2 (second thermal cycle reaction) on the amplification performance of the developed kit. As shown in Supplementary Figure S2, full profile of 32 RM Y-STRs could be observed at different annealing temperatures. Whereas, some extra peaks were observed when the annealing temperature was 2°C and 1°C lower than the recommended annealing temperatures. Even so, these non-specific amplification products exerted little influence on allele detection. It should be noted that these artificial peaks may bring about some difficulties when evaluating mixture samples. From the above results, 58.5°C and 57°C were the optimal annealing temperatures for two different PCR cycles, respectively.

Forensic researchers often increase the number of cycles to improve detection rate of STRs in some trace samples. Here, we evaluated the effect of different cycle numbers on the Y-STR profile. As shown in Supplementary Figure S3, the allelic profile of 32 RM Y-STRs could be obtained at different cycle numbers. Besides, it was expected that peak height increased with increasing cycle numbers. Given that a better peak height balance was observed at 30 cycles, we proposed that the best cycle number was 30.

The concentration of PCR reagents may influence the multiplex amplification performance. It is critical to assess the effect of fluctuation of the PCR reagent concentrations on the overall appearance of Y-STRs. For the primer mix, all profile of 32 RM Y-STRs could be observed at various concentrations of primer mix (Supplementary Figure S4). Furthermore, no extra peaks were detected in the read region (90–490 bp), and the intra-color and inter-color balances were always greater than 70% and 50% at different concentrations of the primer mix, respectively. For reaction mix and C-Taq polymerase, similar phenomena were observed (Supplementary Figures S5, S6). Overall, the multiplex amplification system comprising 32 RM Y-STRs showed robust amplification and tolerated variations in PCR reagent concentrations.

### Accuracy and Precision Studies

In this study, three different researchers amplified and analyzed the 9948 DNA sample using the developed kit. Consistent genotype results for the 32 RM Y-STRs were obtained.

There are eight overlapping Y-STR loci between the system in this study and the AGCU Y37 kit. Therefore, 10 samples were randomly selected to evaluate the concordances of these RM Y-STRs. We found that overlapped RM Y-STR loci between two kits showed the same allelic profile for the same sample, implying that the kit exhibited good concordances.

**FIGURE 2:**
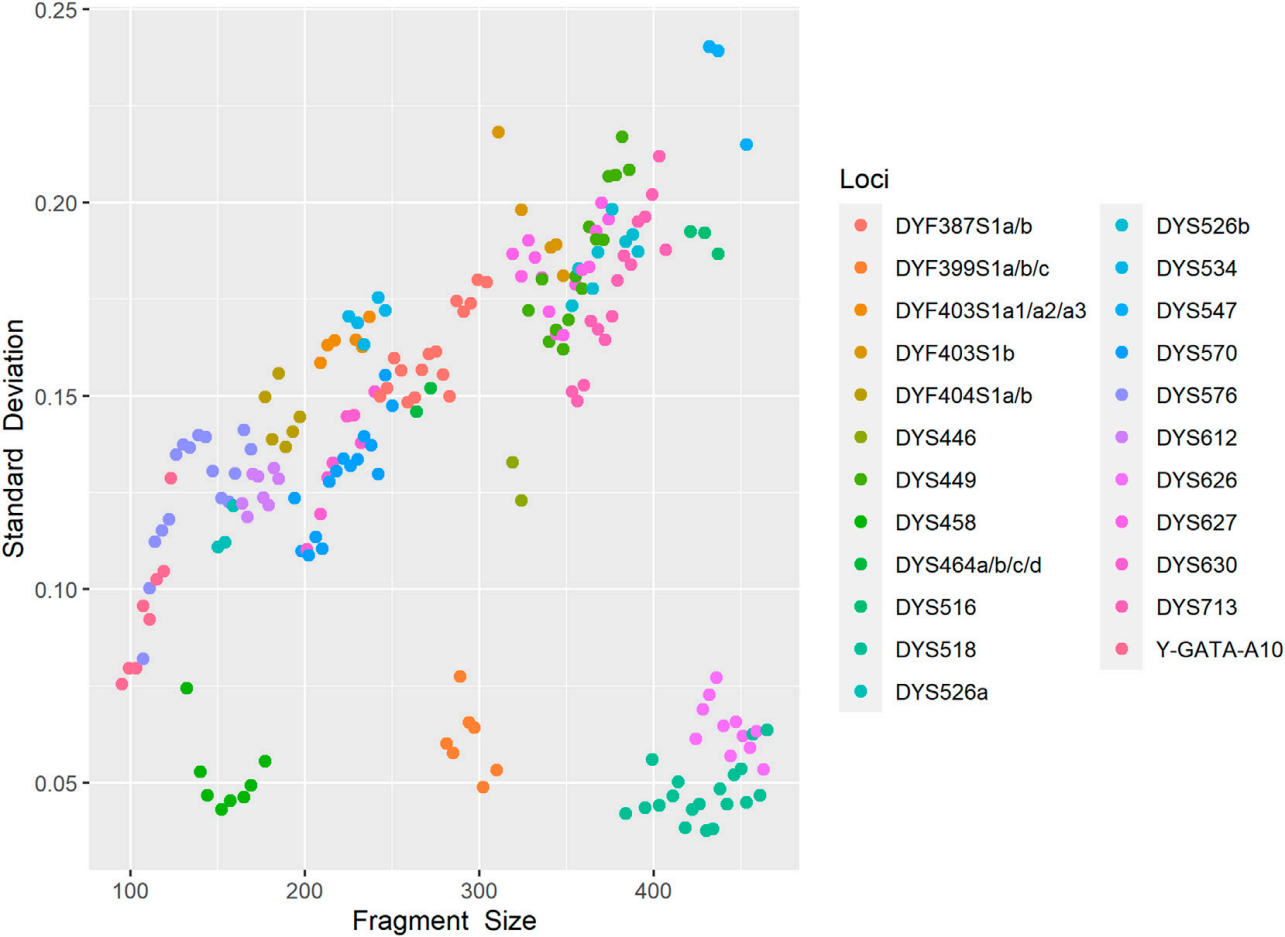
Fragment size scoring variation for control allelic ladder of each locus detected on the 3500xL Genetic Analyzer in 24 replicated runs. Each point represents the standard errors of estimated fragment size for each locus.

Allele size accuracy is the indispensable prerequisite for obtaining a credible genetic profile. We injected an allelic ladder into the 24 capillaries on the 3500xL Genetic Analyzer to assess fluctuations in allele size. As shown in Figure 2, standard variations of each allele ranged from 0.0370 to 0.2400, implying a relatively small allele size variation. The results indicated that the developed kit achieved sufficient genotyping accuracy and size precision.

### Sample Compatibility

Forensic workers often use different bloodstain cards to store samples. It is of great importance to evaluate influence of different storage cards on the overall performance of the kit. Here, we amplified and analyzed bloodstain samples deposited in 11 types of commercially available storage cards (FTA, Haoyuan, Kecaifeng reinforce, Kecaifeng mini, Dabo, Xinhai, Xinhai 503, filter paper, Bokun classic, Bokun reinforce, and Bokun mini). We found that samples on bloodstain cards from the same individual showed a consistent allelic profile of 32 RM Y-STRs, indicating the kit possessed good sample adaptability.

We also assessed the power of the kit to detect case-type samples. The complete allelic profile of 32 RM Y-STR loci was obtained from simulated samples (hair root, blood stain on walls, saliva stains from table top, semen stains from underpants, and cell phone swabs). The allelic profile of a hair root sample from one individual for the 32 RM Y-STRs is shown in Supplementary Figure S7. More importantly, biological samples from the same donor produced identical allelic profiles for the 32 RM Y-STRs. Therefore, we proposed that the developed system could be utilized to detect common case-type samples.

### Mixture Studies

Mixed samples are frequently encountered in sexual assault cases. Further, mixed samples also result from occasional contaminations from operator handling or from the workplace. Therefore, it is necessary to evaluate the power of the kit to discriminate DNA mixtures. Firstly, we mixed 9948 and 9947A samples at 19:1, 9:1, 3:1, 1:1, 1:3, 1:9, and 1:19 ratios and amplified these mixtures using the developed kit. As shown in Supplementary Figure S8, the full allelic profile of the male contributor was observed at 1:1, 1:3, 1:9, and 1:19 ratios. For male–male mixture, we constructed mixtures of two unrelated males in 19:1, 9:1, 3:1, 1:1, 1:3, 1:9, and 1:19 ratios. Similarly, a complete allelic profile of a minor contributor could be obtained in any mixed ratios (Supplementary Figure S9). In summary, the kit showed relatively a relatively high discrimination efficiency for mixed samples.

### Sensitivity Studies

**FIGURE 3:**
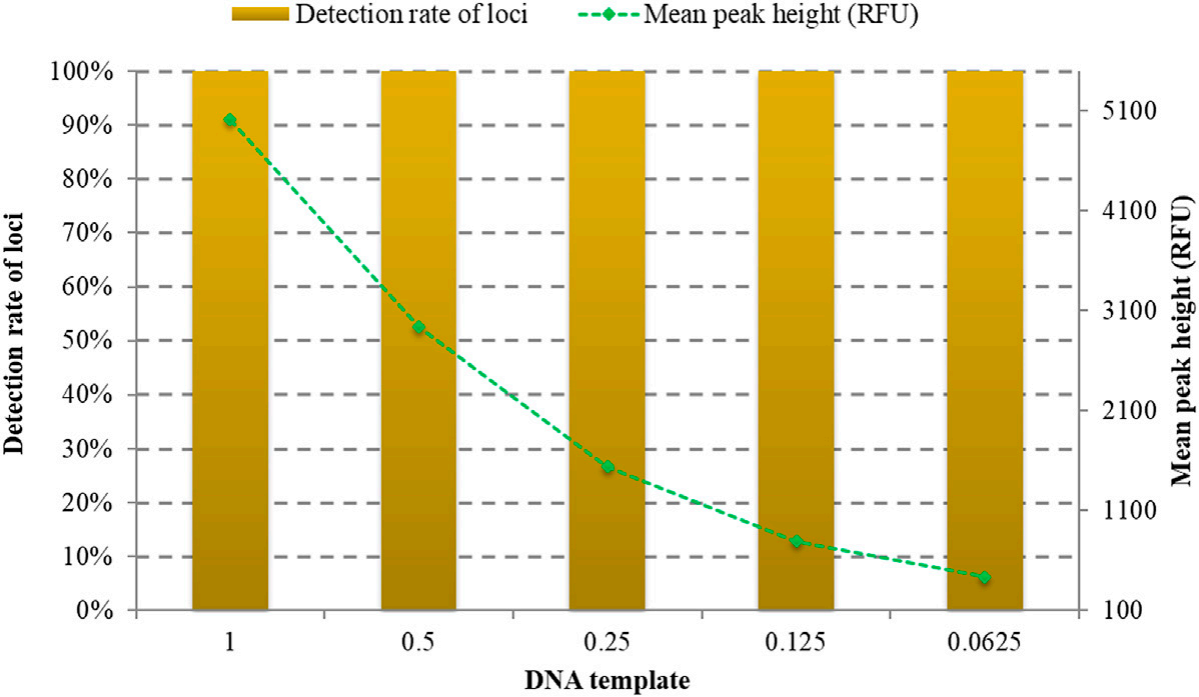
Allele detection rates and peak heights of different quantities of the positive DNA sample 9948 amplified by the developed kit.

Biological samples collected from forensic scenes may contain trace amounts of DNA. Different quantities of DNA samples were tested to assess the detection limitation of the kit. As shown in Figure 3, full allelic profile of 32 RM Y-STRs could be observed at 1, 0.5, 0.25, 0.125, and 0.0625 ng. What is more, the peak height gradually decreased with the reduction of DNA amount. Therefore, the recommended quantity of DNA for the developed RM Y-STRs system is 1 ng.

### Species Specificity

Common animals sometimes exist in crime scenes. Thus, it is important to evaluate the species specificity of the kit. No cross-reactions of 32 RM Y-STRs were observed for seven common species tested (Supplementary Figure S10). Thus, we proposed that the kit exhibited an unexceptionable species specificity.

### Stability Study

**FIGURE 4:**
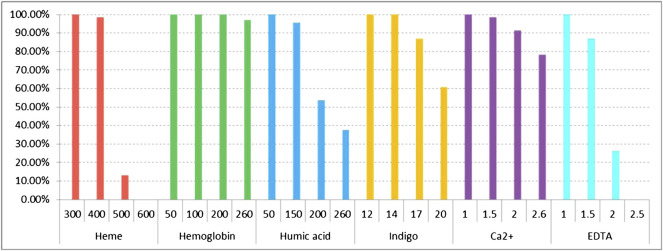
Detection ratios of 32 RM Y-STRs in the presence of different concentrations of PCR inhibitors heme, hemoglobin, humic acid, indigo, Ca^2+^, and EDTA by the HomyGene RM Y32 panel.

There are some PCR inhibitors that commonly exist in forensic biological samples. These inhibitors may interfere with the performance of multiplex amplification. In this study, we explored the tolerance of the kit to frequent inhibitors (heme, hemoglobin, humic acid, indigo, Ca^2+^, and EDTA). As shown in Figure 4 and Supplementary Figure S11, the complete allelic profile of 32 RM Y-STRs could be obtained even with the addition of 300 μM heme. When the concentration of heme increased to 400 and 500 μM, the peak height of larger amplicons decreased and most loci began ceased to be detected. Besides, no peaks were observed with 600 μM heme. With regard to PCR inhibition by hemoglobin, 32 RM Y-STRs showed complete allelic profile at 50 and 100 μM. When the concentration of hemoglobin increased to 200 μM, peak height began to decrease. Moreover, 3% of the 32 RM Y-STR loci were undetected at 260 μM hemoglobin (Figure 4 and Supplementary Figure S12). For humic acid, the large amplicons were missed at 150 ng/μl, and more alleles were undetected when humic acid increased to 200 and 260 ng/μl (Figure 4 and Supplementary Figure S13). With regard to indigo, the complete allelic profile of 32 RM Y-STRs were observed at 12 and 14 mM. However, when the indigo concentration increased to 17 mM, some large amplicons were undetected (Figure 4 and Supplementary Figure S14). For Ca^2+^, the allele peak height gradually decreased with increasing Ca^2+^ concentration (Figure 4 and Supplementary Figure S15). In addition, some alleles were undetected starting at 1.5 mM and only 78.26% of RM Y-STRs were observed at 2.6 mM. For EDTA, the full allelic profile was observed at 1.0 mM, whereas no profile was observed at 2.5 mM (Figure 4 and Supplementary Figure S16). Overall, the developed kit showed relatively good tolerance in the presence of these common inhibitors.

### Population Study

**FIGURE 5:**
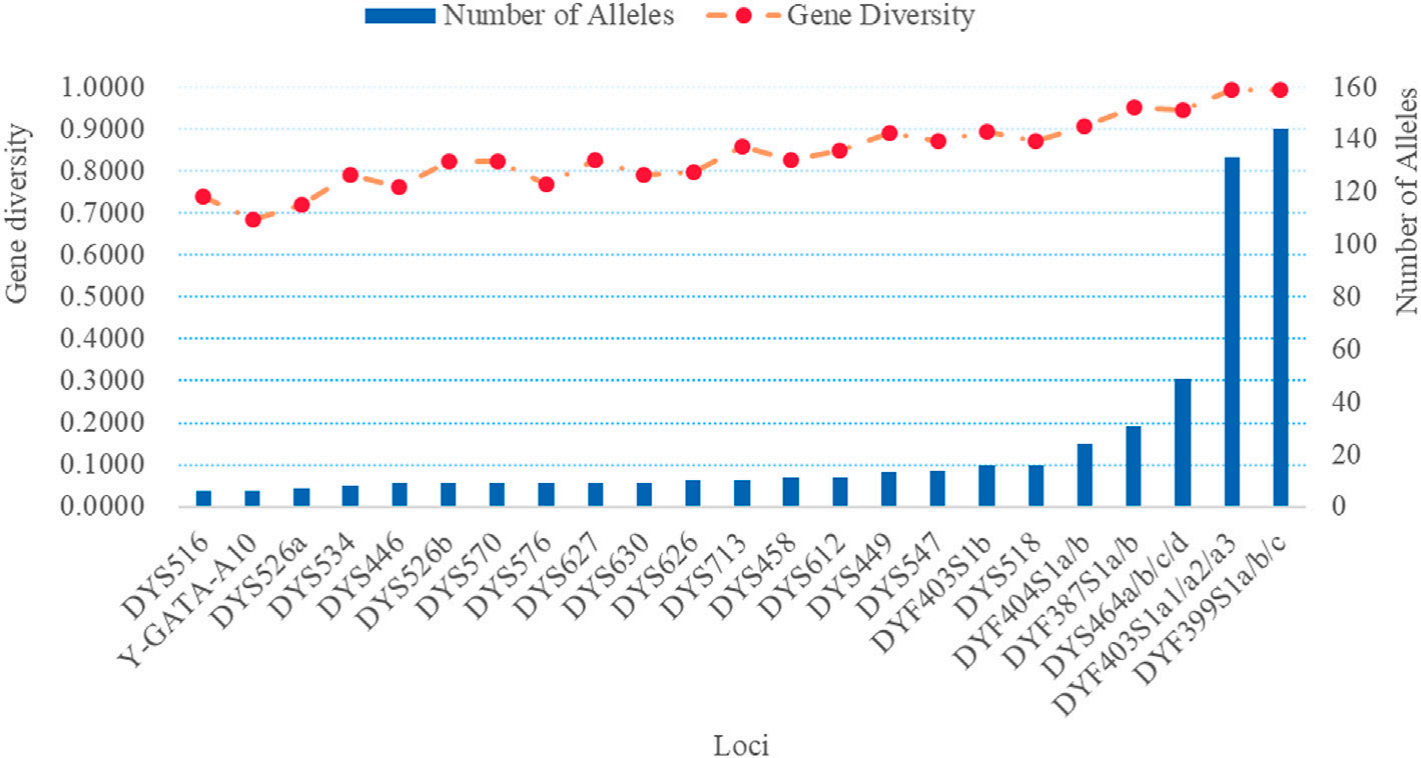
The number of alleles and gene diversities of 32 RM Y-STRs in the Guizhou Han population.

We investigated the genetic distributions and GD of 32 RM Y-STRs in the Guizhou Han population. The genetic profile and allelic frequencies of 32 RM Y-STRs in the Guizhou Han population are given in Supplementary Tables S1, S2, respectively. Furthermore, the number of alleles and GD values of 32 RM Y-STRs observed in the Guizhou Han population are also illustrated in Figure 5. The number of alleles for these 32 RM Y-STRs ranged from 6 (DYS516 and Y-GATA-A10) to 144 (DYF399S1a/b/c). The GD values of these RM Y-STRs distributed from 0.6828 (Y-GATA-A10) to 0.9942 (DYF399S1a/b/c). In a previous study, Chen et al. evaluated the forensic values of the 23 Y-STRs in the Guizhou Han population and found that four loci possessed low GD values (<0.5) (Chen et al., 2018). In the present study, the GD values of 32 RM Y-STRs were greater than 0.5, indicating that they exhibited relatively high genetic diversities in the GuiZhou Han population. What is more, no shared haplotype was observed for these 32 RM Y-STRs in 232 Guizhou Han individuals. The HMP, DC, and HD values of 32 RM Y-STRs in the Guizhou Han population were 0.0043, 1, and 1, respectively. Overall, these 32 RM Y-STRs showed high genetic polymorphisms in the Guizhou Han population and could be used to differentiate unrelated males.

## Conclusion

In the present study, a multiplex amplification panel able to co-amplify 32 RM Y-STRs was developed. The validation studies of the panel revealed that it showed high sensitivity, unexceptionable species specificity, and good tolerance to inhibitors. In addition, the panel was not only suitable for different bloodstain storage cards but also contributed to disentangling mixed samples. Nonetheless, mutation rates of these 32 RM Y-STRs warrant further investigate in the future.

## Data Availability

The original contributions presented in the study are included in the article/Supplementary Material. Further inquiries can be directed to the corresponding author.
