# Supplementary material for: Developmental Validation of a Rapidly Mutating Y-STR Panel Labeled by Six Fluoresceins for Forensic Research
Source: Front Genet. 2022 Mar 3;13:777440. doi: 10.3389/fgene.2022.777440 (PMC8927084; doi:10.3389/fgene.2022.777440)
Supplement: Supplementary file 1 [file DataSheet1.ZIP › Supplementary Materials/Figure legend.docx]

**Supplementary Figure 1.** Amplification length ranges and labeled dyes of the 32 RM Y-STRs.

**Supplementary Figure 2.** Allelic profile of the 32 RM Y-STRs of the positive DNA sample 9948 at different annealing temperatures.

**Supplementary Figure 3.** Allelic profile of the 32 RM Y-STRs of the positive DNA sample 9948 at different PCR cycle numbers.

**Supplementary Figure 4.** Allelic profile of the 32 RM Y-STRs of the positive DNA sample 9948 at different concentrations of primer mix.

**Supplementary Figure 5.** Allelic profile of the 32 RM Y-STRs of the positive DNA sample 9948 at different concentrations of reaction mix.

**Supplementary Figure 6.** Allelic profile of the 32 RM Y-STRs of the positive DNA sample 9948 at different concentrations of C-Taq polymerase.

**Supplementary Figure 7.** Allelic profile of a hair root sample from an individual showing the 32 RM Y-STRs.

**Supplementary Figure 8.** Allelic profile of the mixture of 9948 and 9947A at different mixture ratios.

**Supplementary Figure 9.** Allelic profile of the mixture of two unrelated males at different mixture ratios.

**Supplementary Figure 10.** Allelic profile of the 32 RM Y-STRs for seven common animals.

**Supplementary Figure 11.** Allelic profile of the 32 RM Y-STRs for different concentrations of heme.

**Supplementary Figure 12.** Allelic profile of the 32 RM Y-STRs for different concentrations of hemoglobin.

**Supplementary Figure 13.** Allelic profile of the 32 RM Y-STRs for different concentrations of humic acid.

**Supplementary Figure 14.** Allelic profile of the 32 RM Y-STRs for different concentrations of indigo.

**Supplementary Figure 15.** Allelic profile of the 32 RM Y-STRs for different concentrations of Ca^2+^.

**Supplementary Figure 16.** Allelic profile of the 32 RM Y-STRs for different concentrations of EDTA.
