# Supplementary figures and images for: Developmental Validation of a Rapidly Mutating Y-STR Panel Labeled by Six Fluoresceins for Forensic Research
Source: Front Genet. 2022 Mar 3;13:777440. doi: 10.3389/fgene.2022.777440 (PMC8927084; doi:10.3389/fgene.2022.777440)

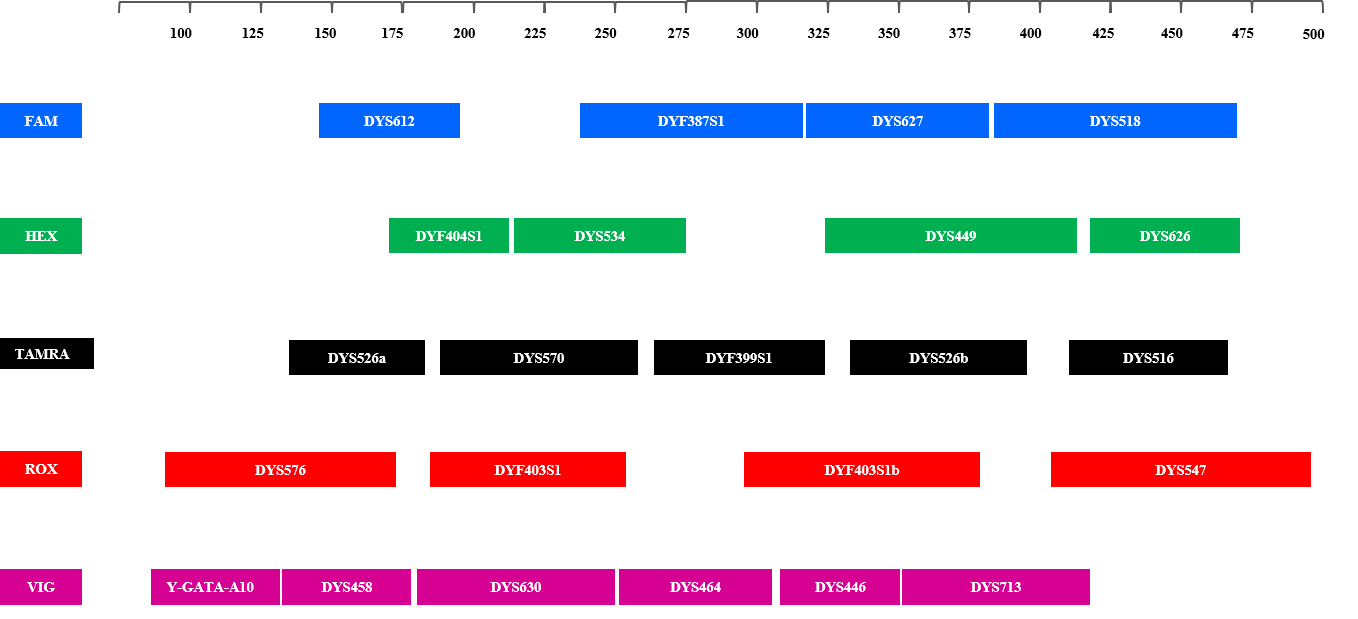

Supplement: Supplementary file 1 [file DataSheet1.ZIP › Supplementary Materials/Supplementary Figure 1.tif]

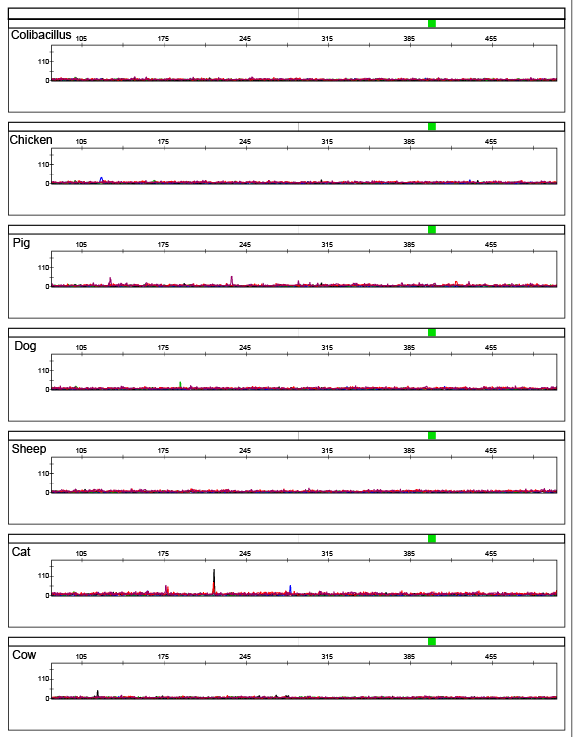

Supplement: Supplementary file 1 [file DataSheet1.ZIP › Supplementary Materials/Supplementary Figure 10.png]

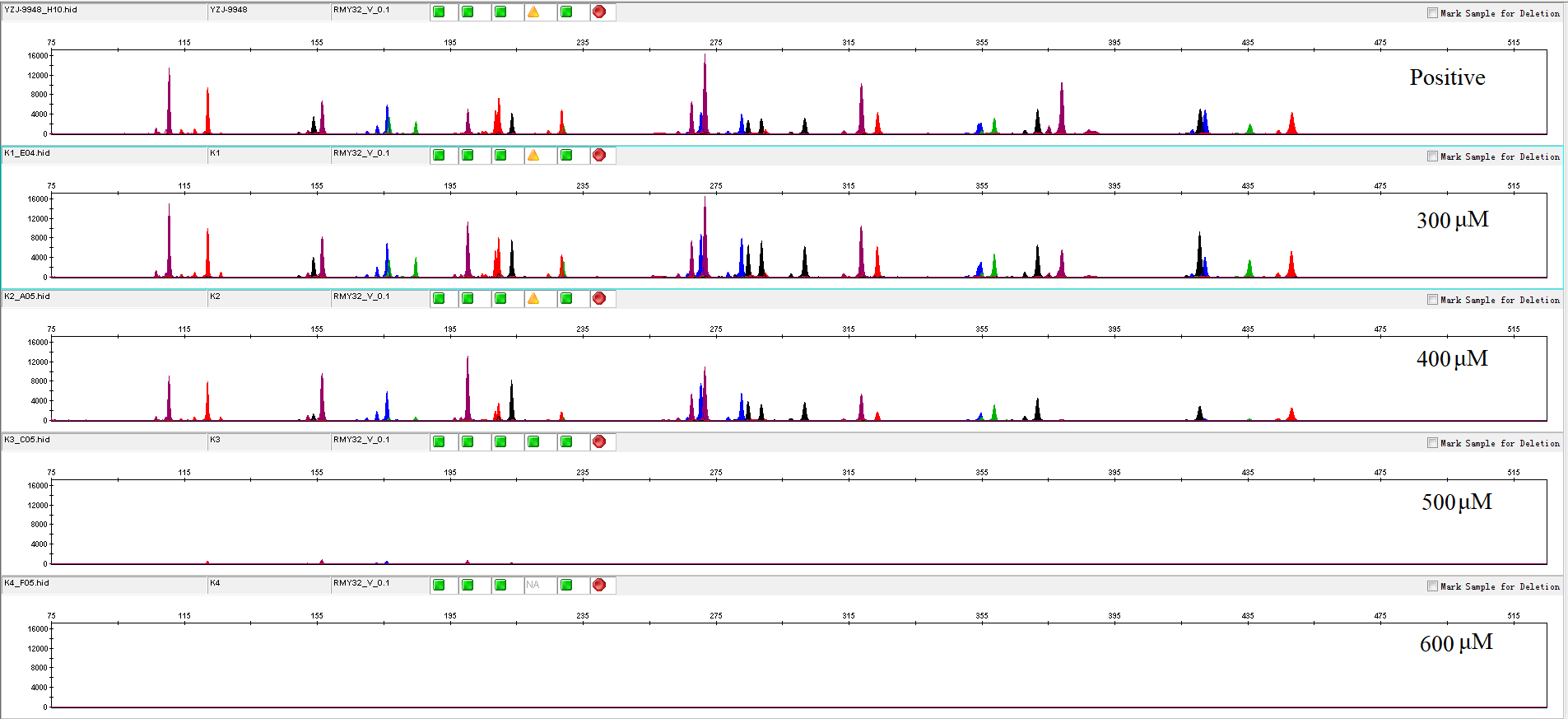

Supplement: Supplementary file 1 [file DataSheet1.ZIP › Supplementary Materials/Supplementary Figure 11.png]

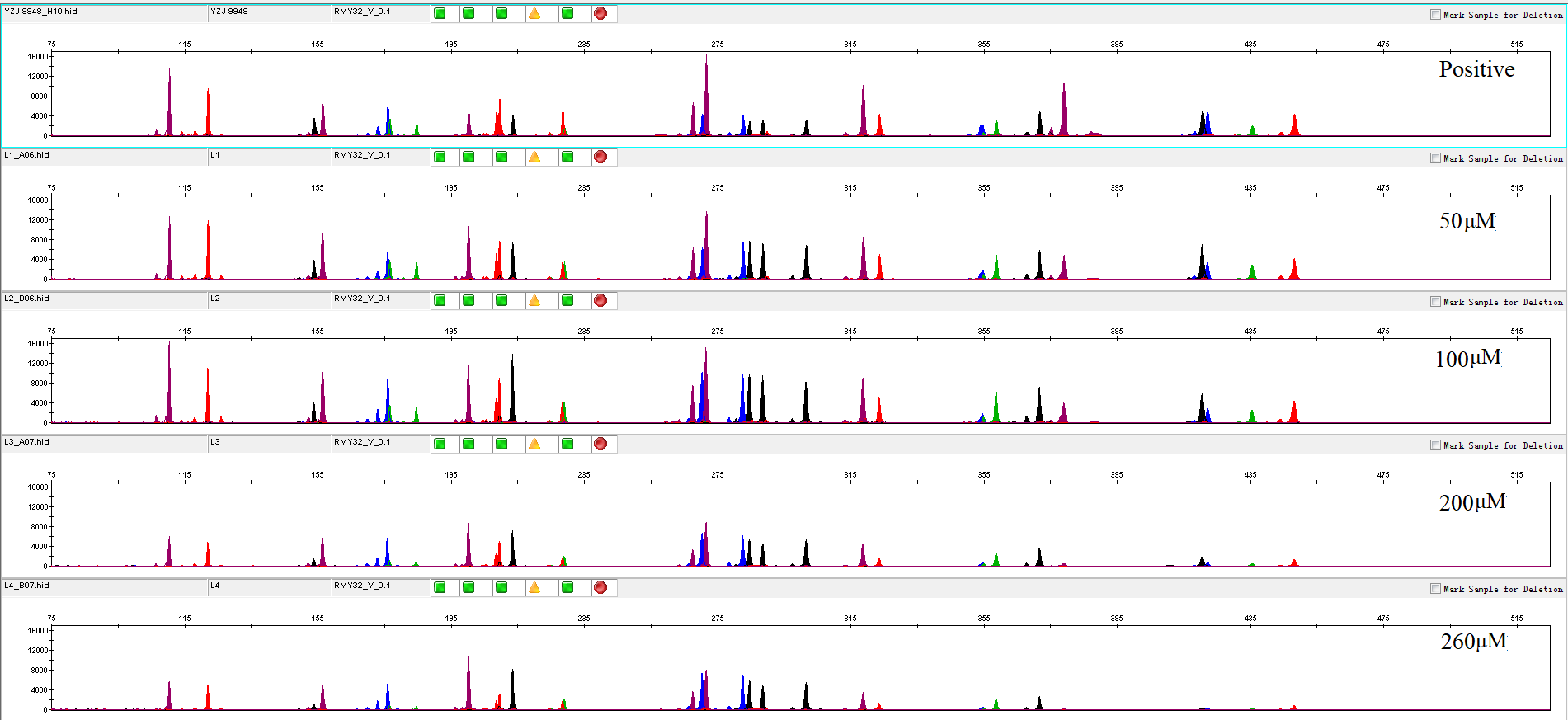

Supplement: Supplementary file 1 [file DataSheet1.ZIP › Supplementary Materials/Supplementary Figure 12.png]

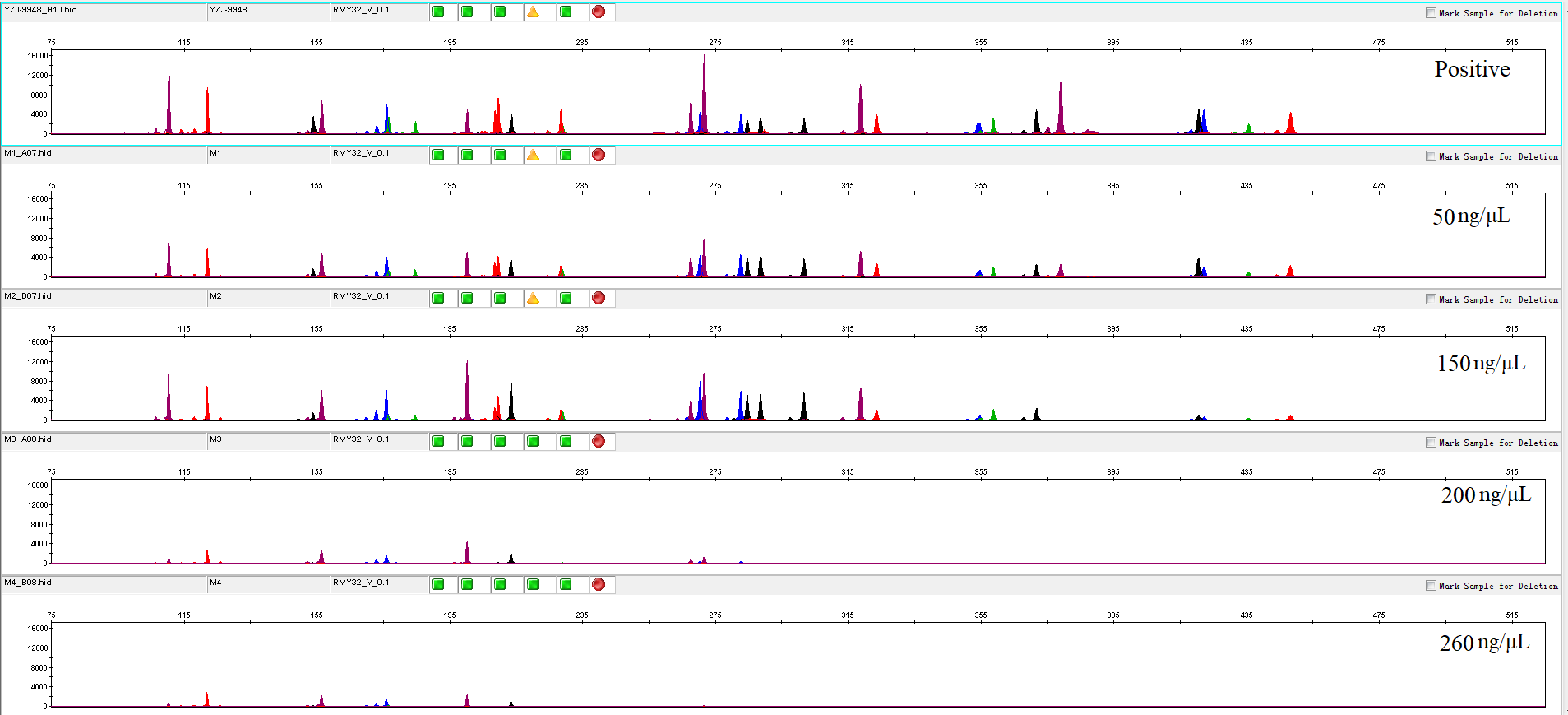

Supplement: Supplementary file 1 [file DataSheet1.ZIP › Supplementary Materials/Supplementary Figure 13.png]

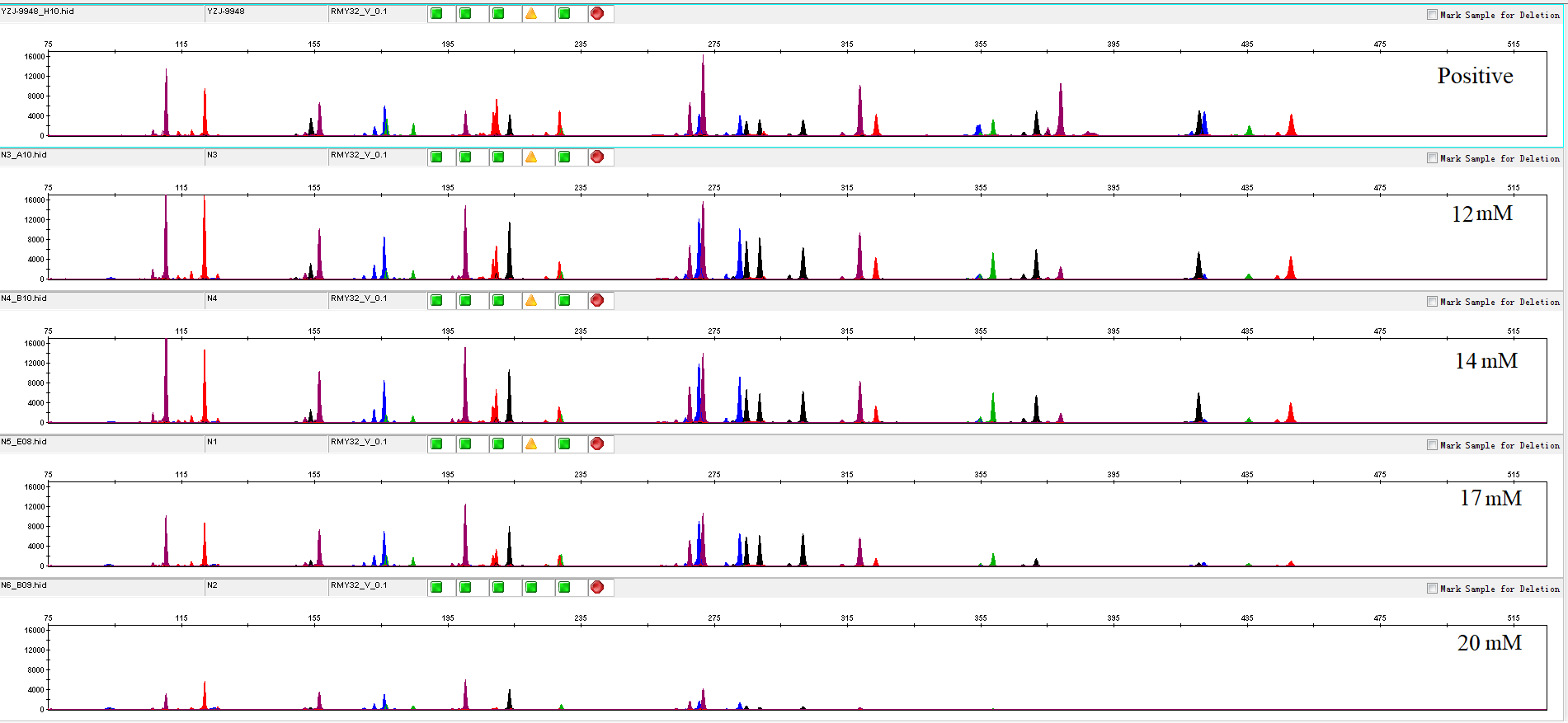

Supplement: Supplementary file 1 [file DataSheet1.ZIP › Supplementary Materials/Supplementary Figure 14.png]

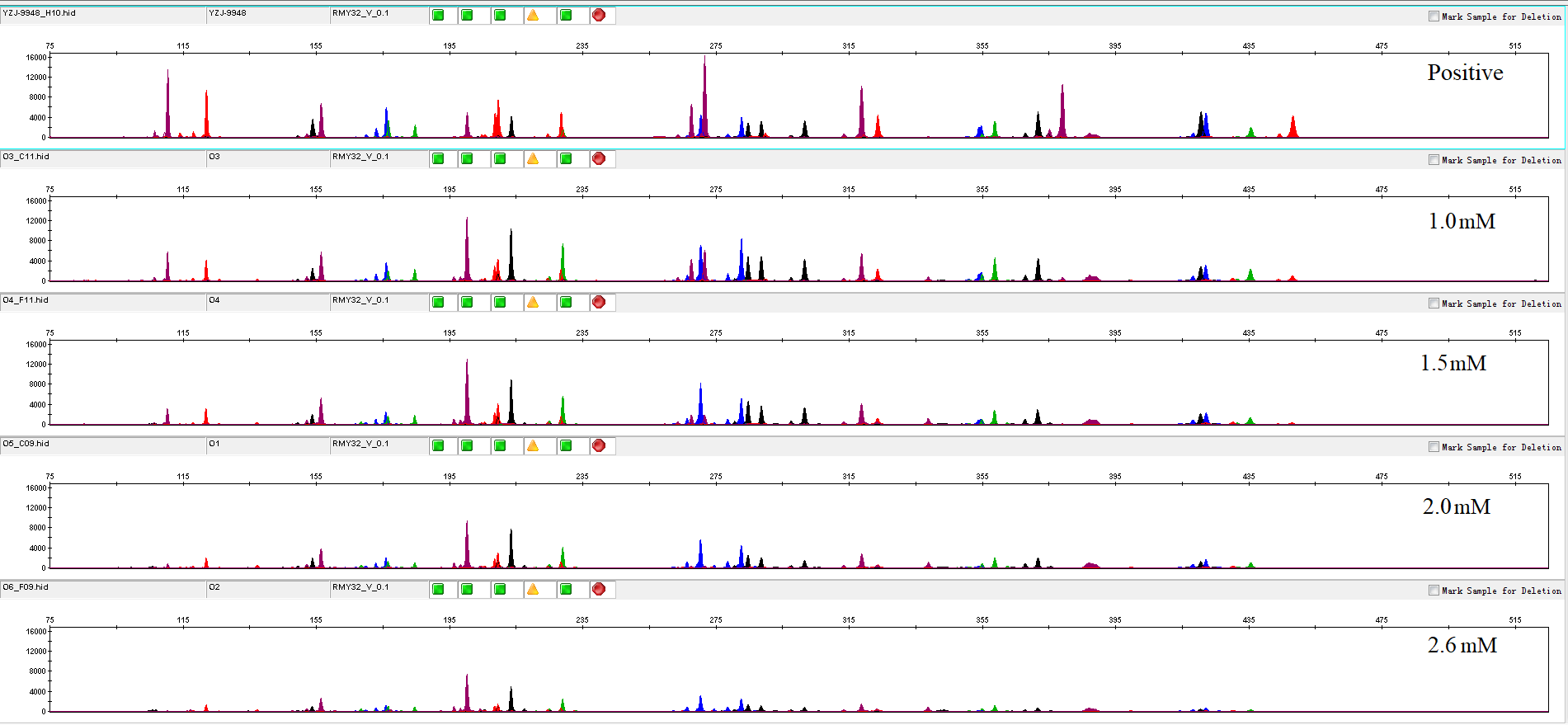

Supplement: Supplementary file 1 [file DataSheet1.ZIP › Supplementary Materials/Supplementary Figure 15.png]

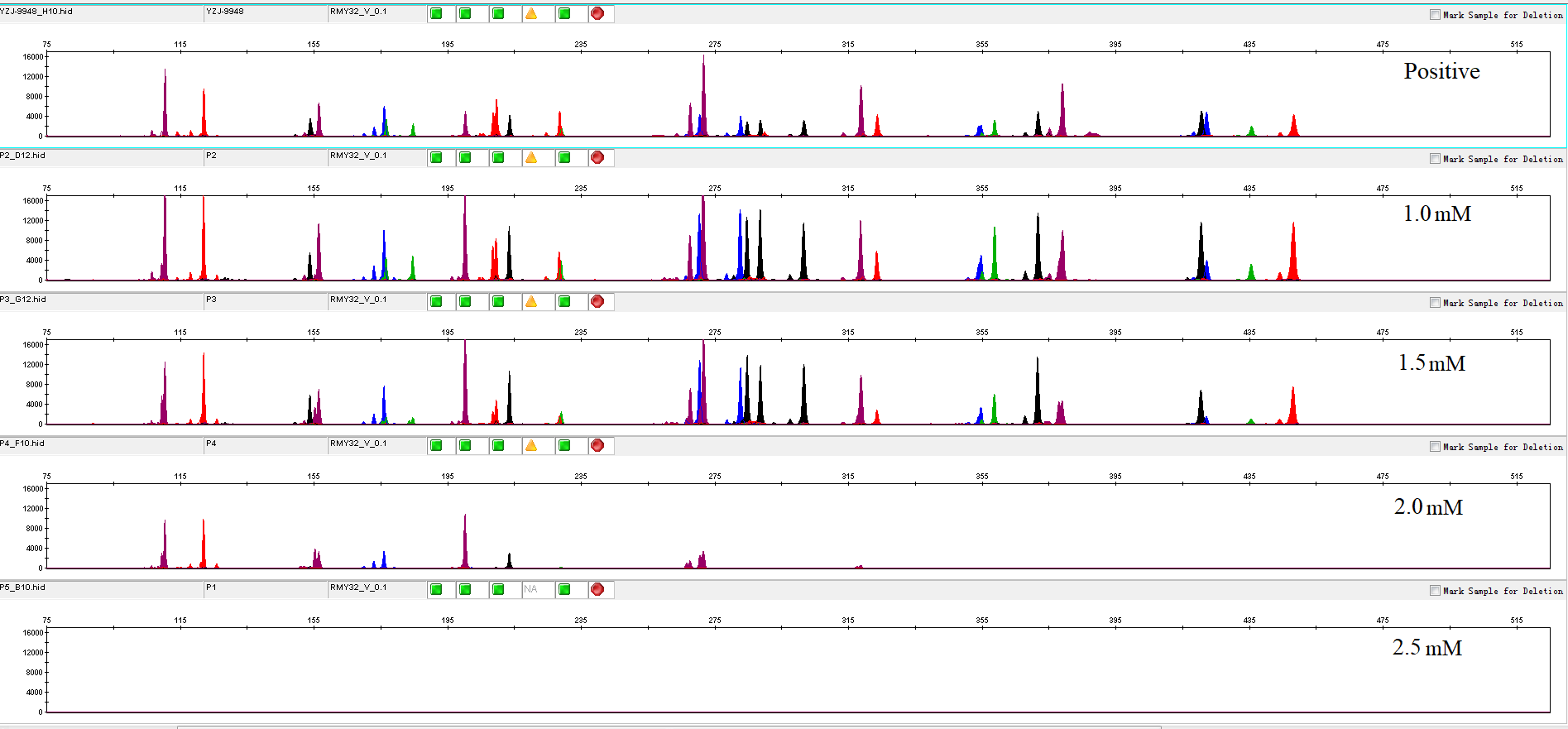

Supplement: Supplementary file 1 [file DataSheet1.ZIP › Supplementary Materials/Supplementary Figure 16.png]

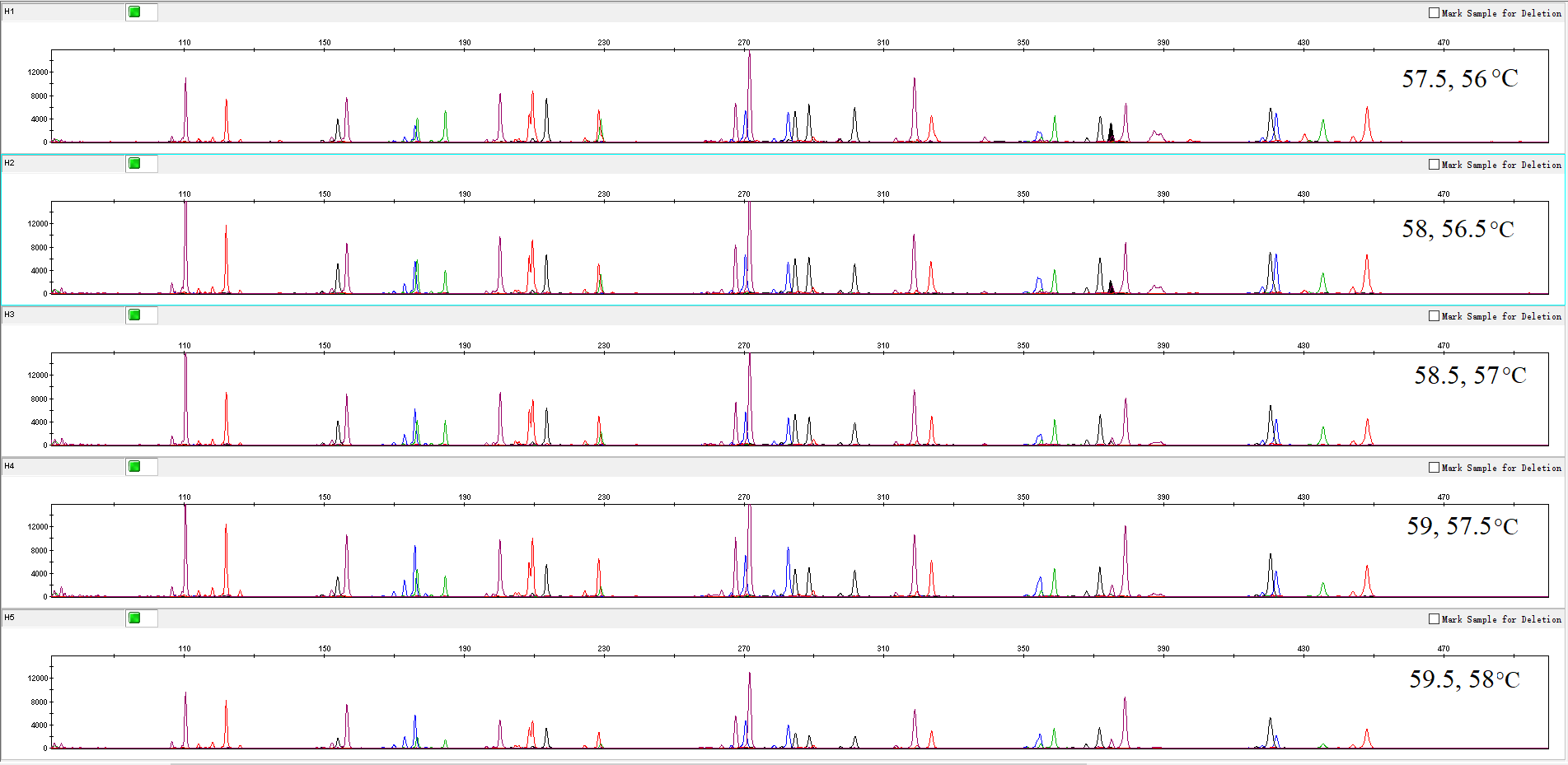

Supplement: Supplementary file 1 [file DataSheet1.ZIP › Supplementary Materials/Supplementary Figure 2.png]

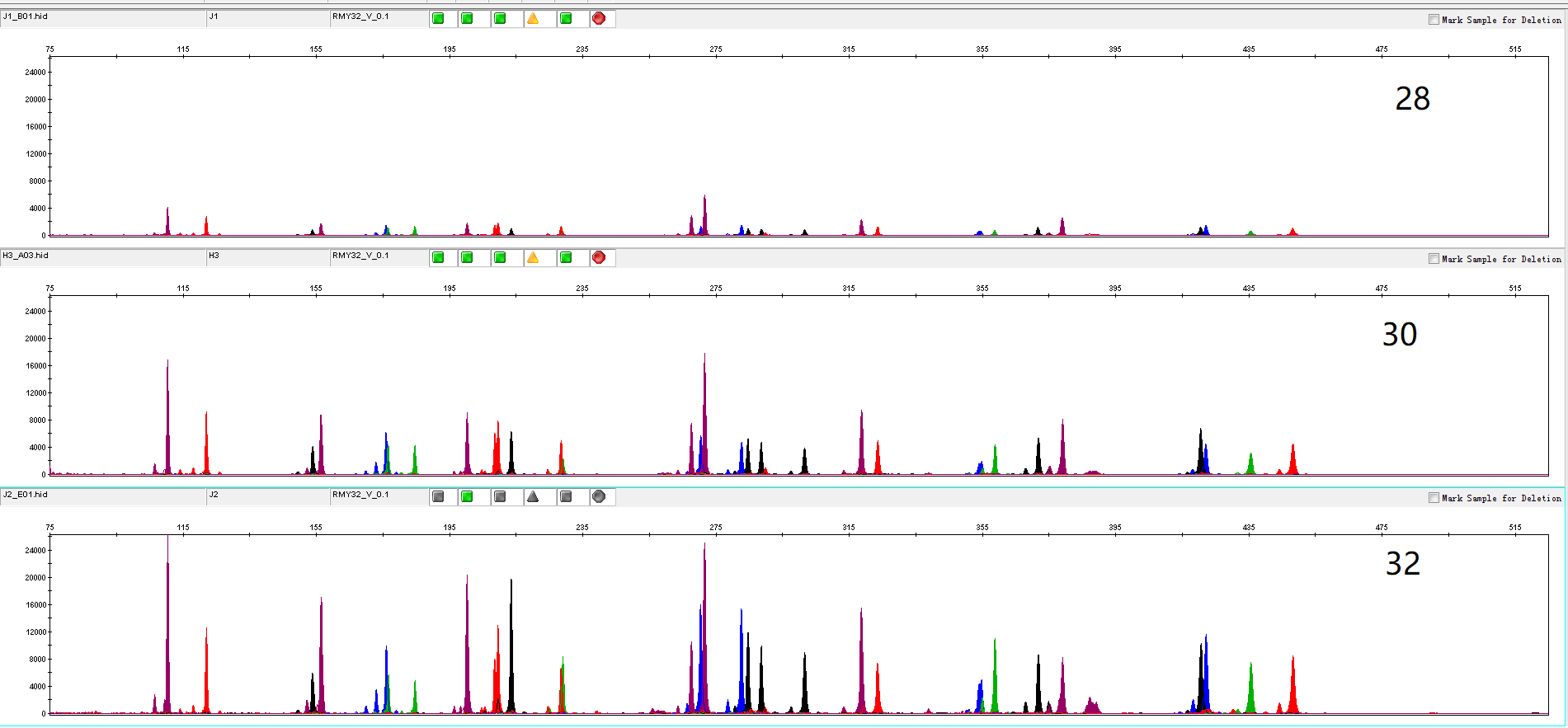

Supplement: Supplementary file 1 [file DataSheet1.ZIP › Supplementary Materials/Supplementary Figure 3.png]

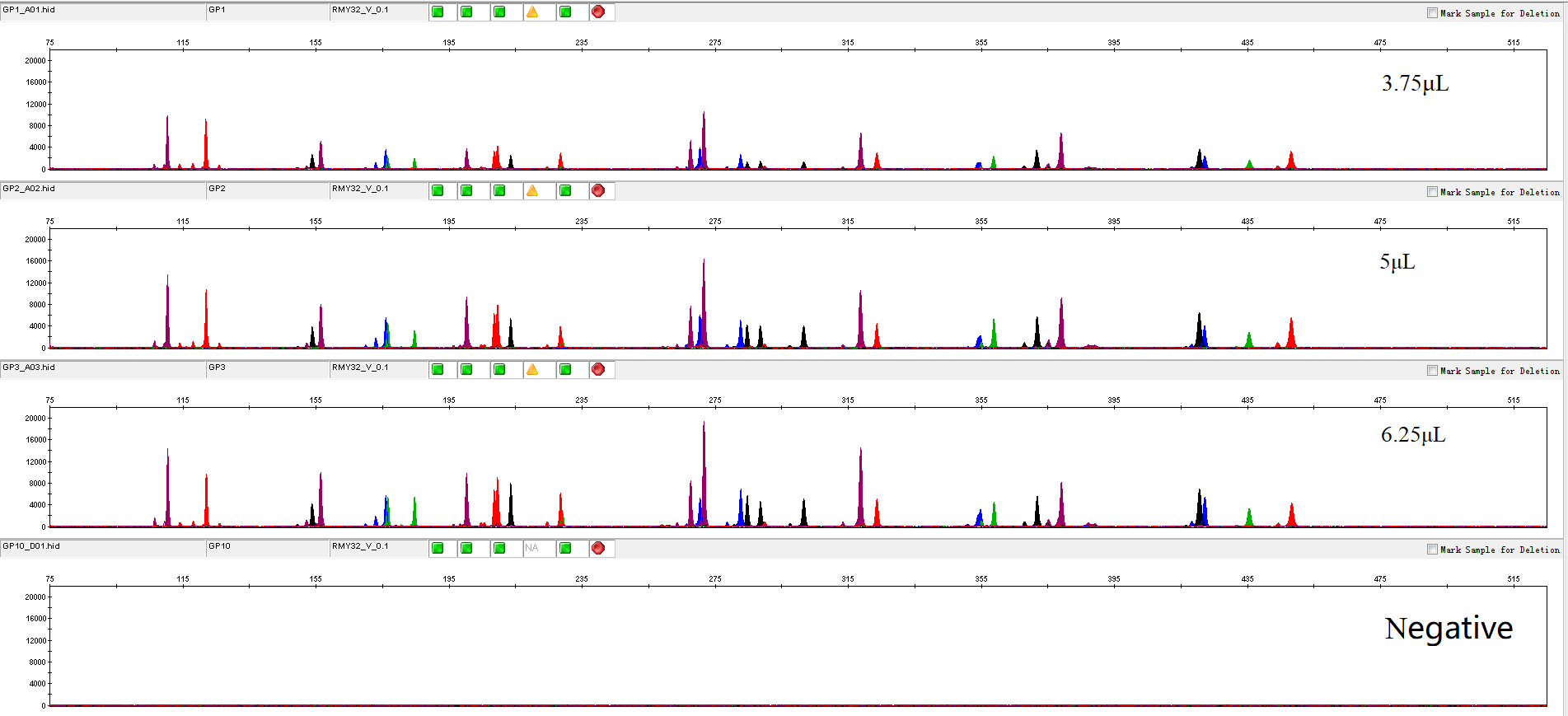

Supplement: Supplementary file 1 [file DataSheet1.ZIP › Supplementary Materials/Supplementary Figure 4.png]

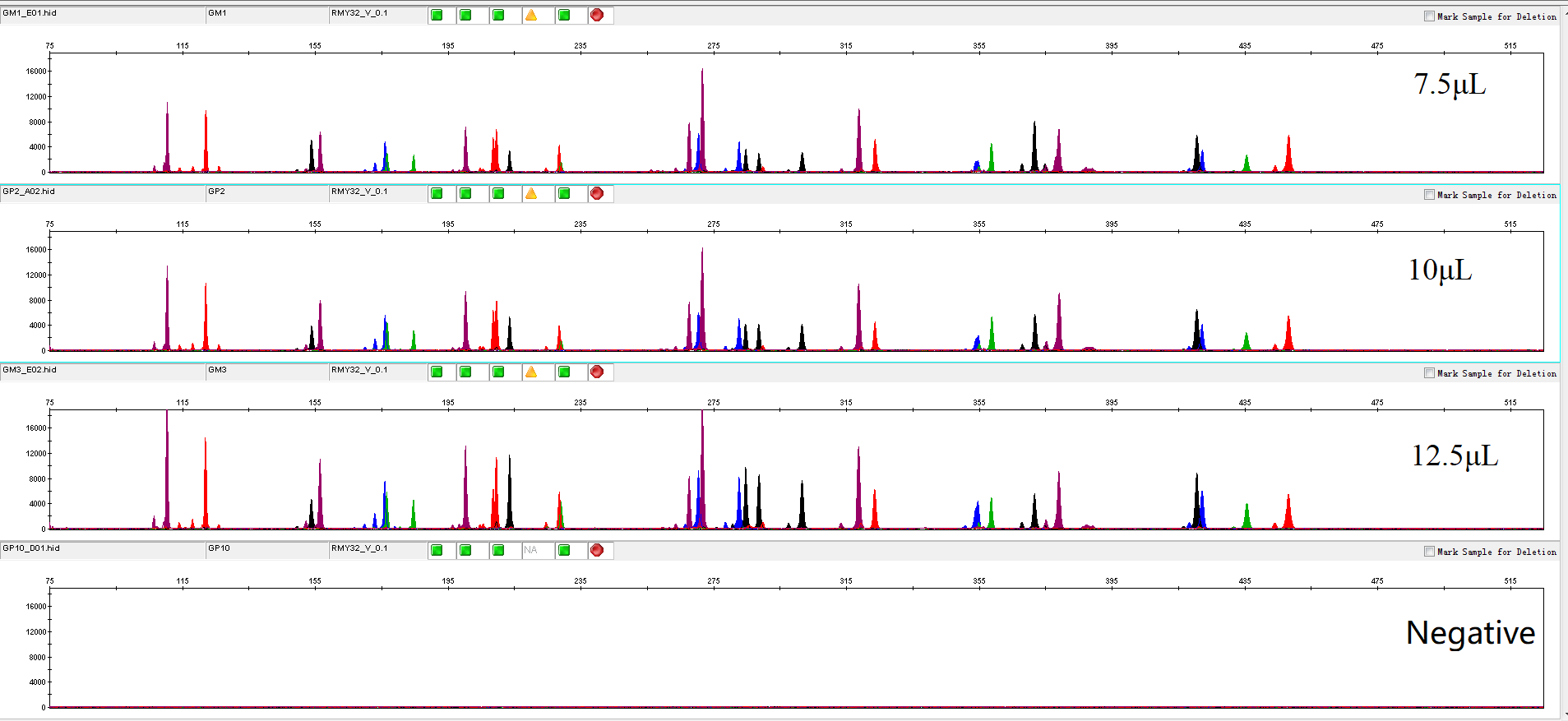

Supplement: Supplementary file 1 [file DataSheet1.ZIP › Supplementary Materials/Supplementary Figure 5.png]

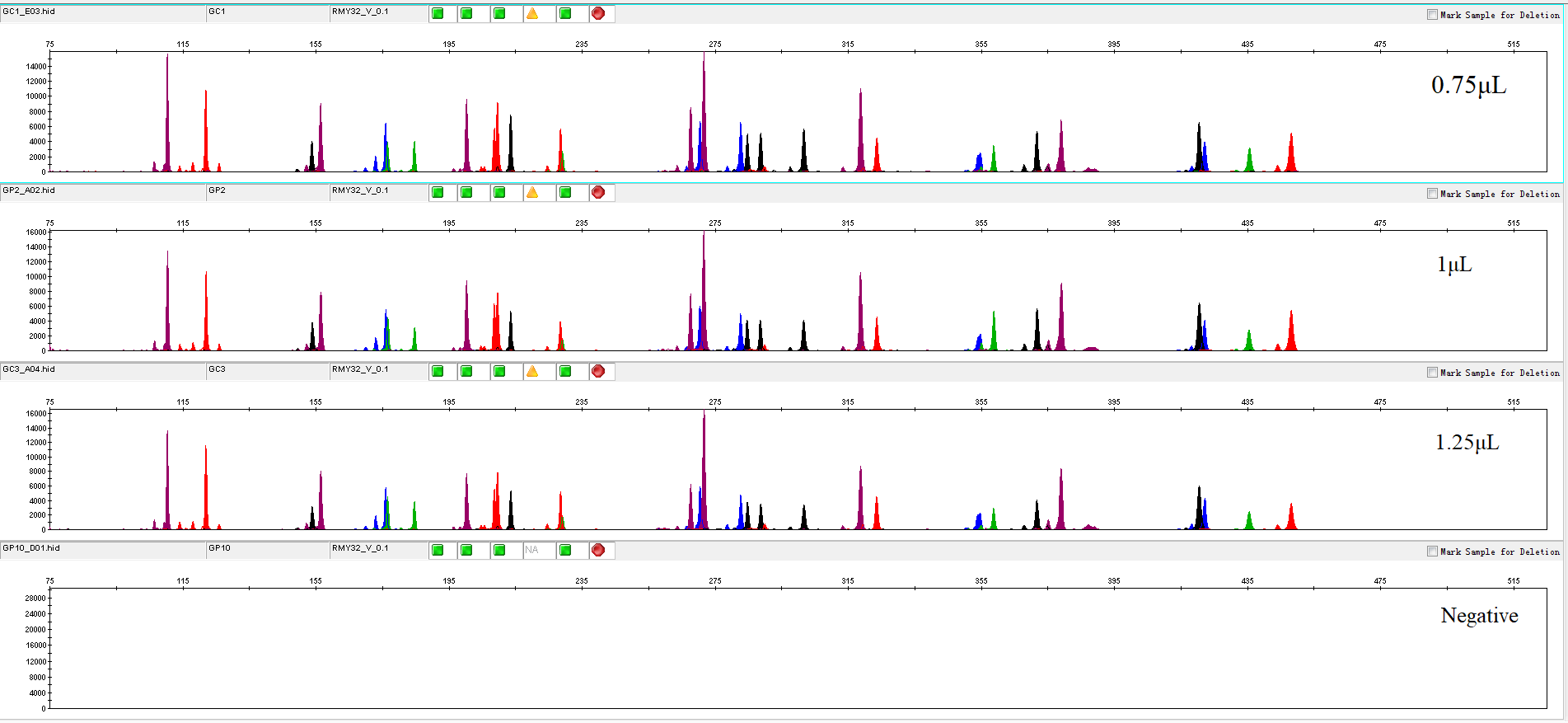

Supplement: Supplementary file 1 [file DataSheet1.ZIP › Supplementary Materials/Supplementary Figure 6.png]

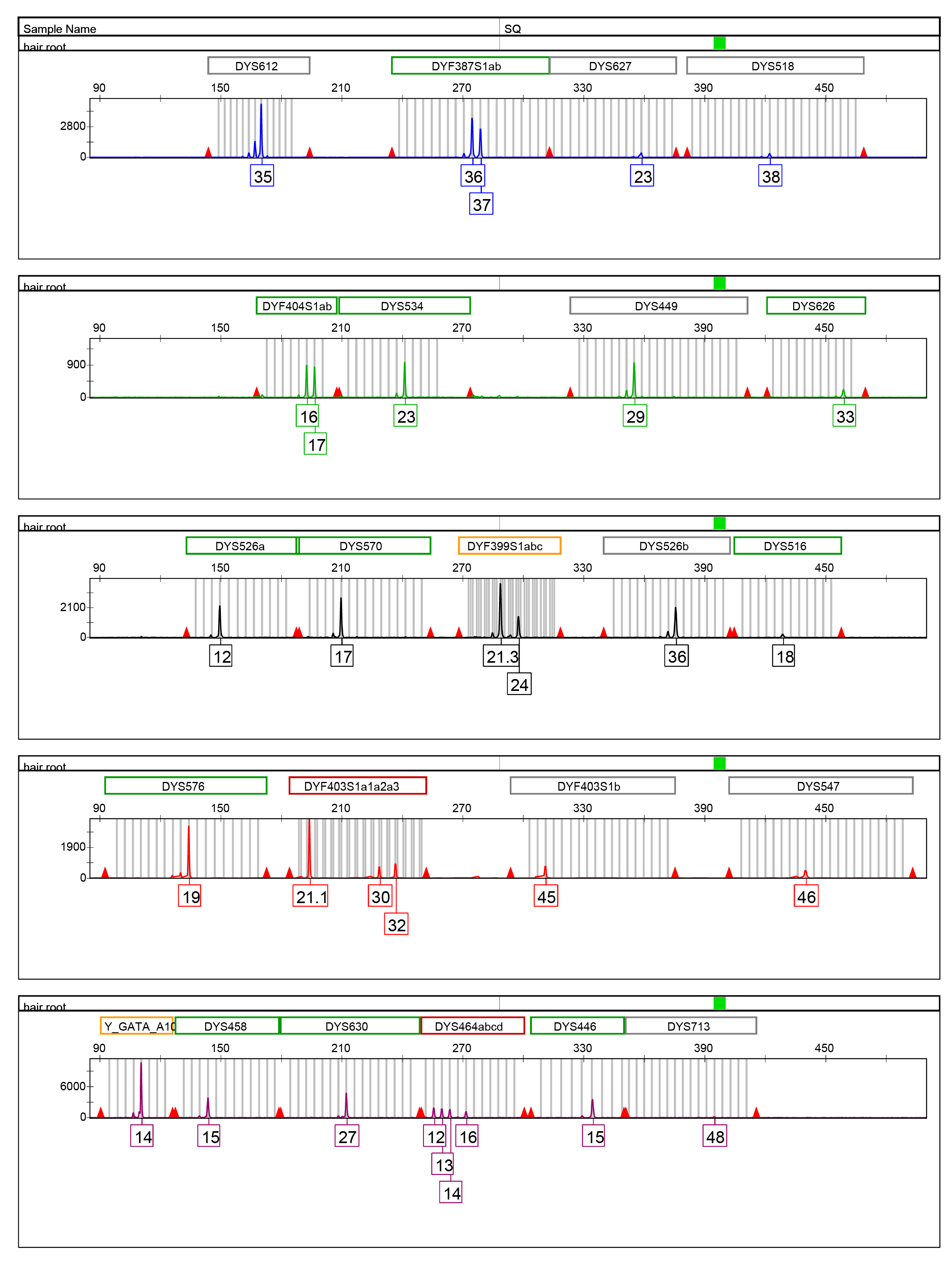

Supplement: Supplementary file 1 [file DataSheet1.ZIP › Supplementary Materials/Supplementary Figure 7.png]

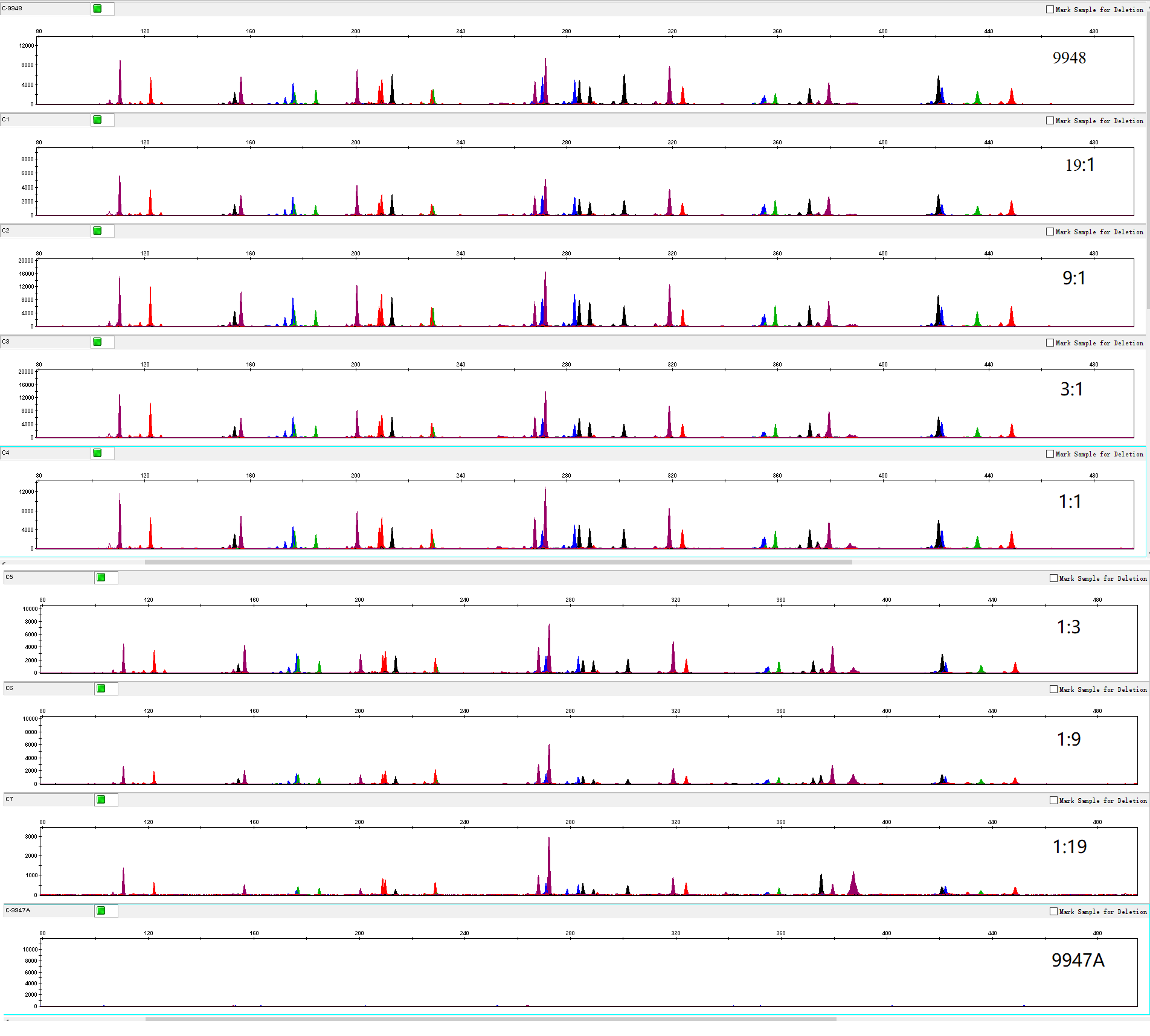

Supplement: Supplementary file 1 [file DataSheet1.ZIP › Supplementary Materials/Supplementary Figure 8.png]

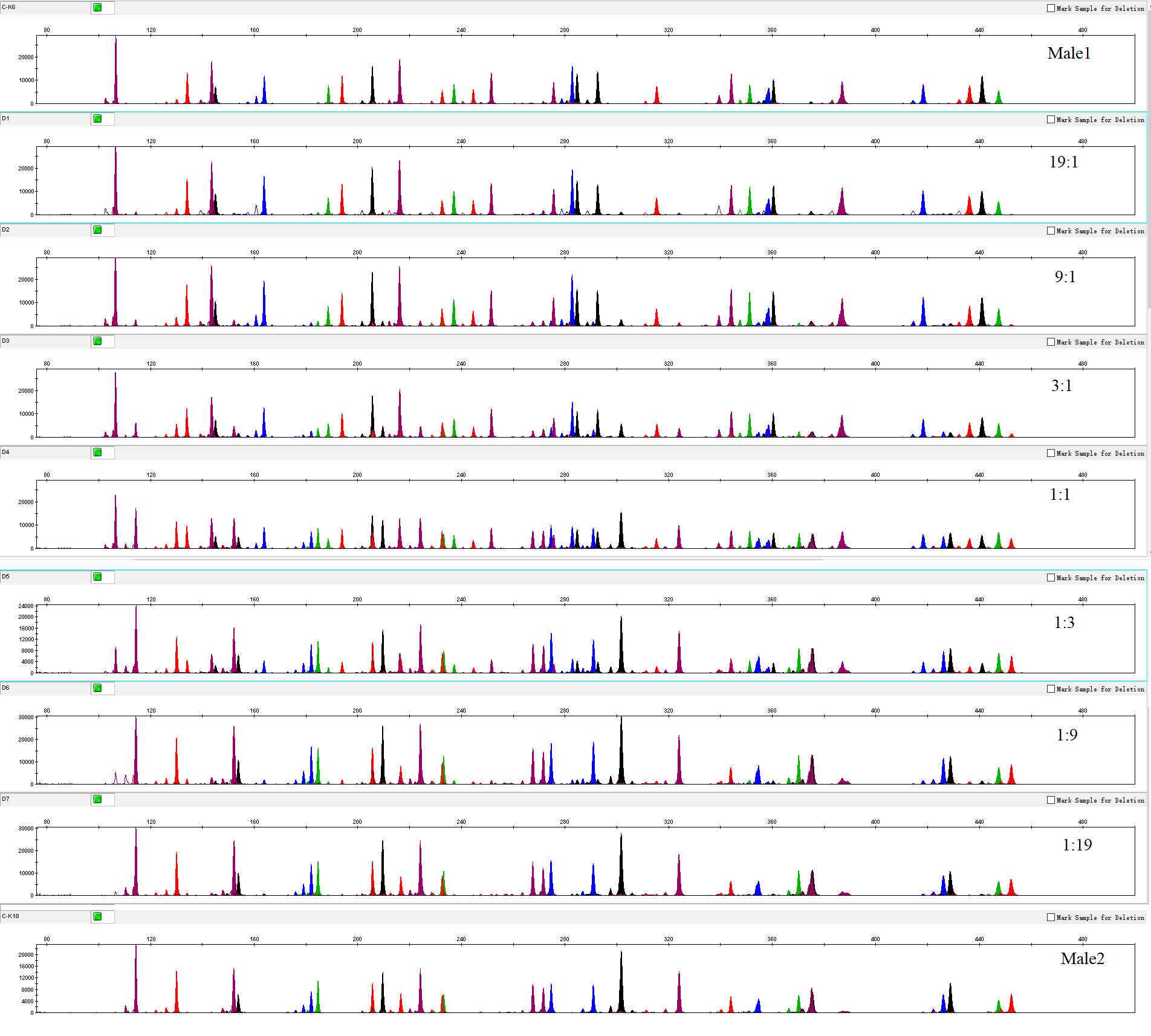

Supplement: Supplementary file 1 [file DataSheet1.ZIP › Supplementary Materials/Supplementary Figure 9.png]
